# Conscientious Objection to Animal Experimentation in Italian Universities

**DOI:** 10.3390/ani7030024

**Published:** 2017-03-13

**Authors:** Ilaria Baldelli, Alma Massaro, Susanna Penco, Anna Maria Bassi, Sara Patuzzo, Rosagemma Ciliberti

**Affiliations:** 1 Dipartimento di Scienze Chirurgiche e Diagnostiche Integrate (DISC), Università di Genova, 16132 Genova, Italy; ilaria.baldelli@unige.it; 2 Unità Operativa di Chirurgia Plastica e Ricostruttiva, IRCCS Azienda Ospedaliera Universitaria San Martino -IST Genova, 16132 Genoa, Italy; 3 Dipartimento di Antichità, Filosofia, Storia, Geografia (DAFIST), Università di Genova, 16126 Genova, Italy; almamassaro@gmail.com; 4 Dipartimento di Medicina Sperimentale (DIMES), Università di Genova, 16132 Genova, Italy; Susanna.Penco@unige.it (S.P.); ambassi@medicina.unige.it (A.M.B.); 5 School of Medicine and Surgery, University of Verona, P.le L. A. Scuro 10, 37134 Verona, Italy; 6 Dipartimento di Scienze della Salute (DISSAL), Università di Genova, 16132 Genova, Italy; rosellaciliberti@yahoo.it

**Keywords:** animal ethics, 3Rs, conscientious objection, veterinary education, science education, non-animal methods

## Abstract

**Simple Summary:**

This paper examines the trend of Italian academic faculties in complying with the obligation to inform university students of their right to exercise their conscientious objection to scientific or educational activities involving animals, hereafter written as “animal CO”, as established by Law 413/1993, “Norme sull’obiezione di coscienza alla sperimentazione animale” (“Rules on conscientious objection to animal experimentation”), thereafter “Law 413/1993”. Despite an increasing interest in the principles of animal ethics by the international community, this law is still largely disregarded more than 20 years after its enactment. The Ethics Committees, Animal Welfare Committees, as well as the Italian Ministry of Education, University and Research should preside over and monitor the Universities’ compliance with the duty to disclose animal CO.

**Abstract:**

In Italy, Law 413/1993 states that public and private Italian Institutions, including academic faculties, are obliged to fully inform workers and students about their right to conscientious objection to scientific or educational activities involving animals, hereafter written as “animal CO”. However, little monitoring on the faculties’ compliance with this law has been performed either by the government or other institutional bodies. Based on this premise, the authors have critically reviewed the existing data and compared them with those emerging from their own investigation to discuss limitations and inconsistencies. The results of this investigation revealed that less than half of Italian academic faculties comply with their duty to inform on animal CO. Non-compliance may substantially affect the right of students to make ethical choices in the field of animal ethics and undermines the fundamental right to express their own freedom of thought. The Italian Ministry of Education, Universities and Research, ethics committees and animal welfare bodies should cooperate to make faculties respect this law. Further research is needed to better understand the reasons for the current trend, as well as to promote the enforcement of Law 413/1993 with particular regard to information on animal CO.

## 1. Introduction

An increased awareness of the ethical issues relating to research involving animals can be found in the Italian Law 413/1993 [1], originally referred to as Law 116/1992, “Attuazione della direttiva CEE n. 609/86 in materia di protezione degli animali utilizzati a fini sperimentali o ad altri fini scientifici” (“Implementation of the Directive CEE n. 609/86 on the protection of animals for experimental or scientific purposes”), now replaced by Law 26/2014, “Attuazione della direttiva 2010/63/UE sulla protezione degli animali utilizzati a fini scientifici” (“Implementation of the Directive 2010/63/UE on the protection of animals for scientific purposes”), thereafter “Law 26/2014”. Law 413/1993 introduced the opportunity for physicians, researchers, students and healthcare providers to not take part in experimental research that involved animals by exercising conscientious objection to scientific or educational activities involving animals, hereafter written as “animal CO”. Furthermore, their decision not to take part in such activities would not expose them to possible adverse consequences arising from their refusal to participate in otherwise legally enforceable acts. Workers had the right to perform alternative activities which did not include animals, while retaining the same qualifications and remuneration. Similarly, students who opted for animal CO had the right to receive educational and teaching activities without animals.

It is important to note that the Italian law uses the expression “animal experimentation” with a general meaning that covers all scientific practices, including educational activities involving animals. Therefore, the Italian law is inadequate as it has chosen poor wording, which is not sufficiently descriptive. In the same way, when the Italian law states the right to opt for animal CO, it applies the same inaccurate reference to the general field of “animal experimentation”, implicitly including the specific sector of educational animal use.

Law 413/1993 aims to safeguard personal freedom to express ethical choices, according to fundamental ethical principles and human rights recognized at an international level [2], as stated in the Universal Declaration of Human Rights; the European Convention for the Protection of Human Rights and Fundamental Freedoms; and in the International Covenant on Civil and Political Rights adopted by the United Nations General Assembly.

In this light, in addition to Law 413/1993 on animal CO, Italian legislation provides two other laws that safeguard the right of healthcare professionals to exercise their conscientious objection: Law 194/1978, “Norme per la tutela sociale della maternità e sull’interruzione volontaria della gravidanza” (“Rules for social protection of maternity and on voluntary interruption of pregnancy”) [3]; and Law 40/2004, “Norme in materia di procreazione medicalmente assistita” (“Rules on medically assisted procreation”) [4].

According to Law 413/1993, all public and private entities who are authorized to perform scientific or educational activities must inform all workers and students of their right to exercise animal CO and provide a specific declaration form that can be repealed at any time. However, with specific attention to academic faculties, Law 413/1993 does not provide details about how this information should be communicated, for instance by posting the full text of the law on the faculties’ websites or during lectures with an explanation by professors. Consequently, monitoring of the faculties’ compliance with their information duty appears to be difficult. Currently, a starting control has been conducted by the following bodies and associations.

In 2009, the Italian National Bioethics Committee (NBC), a government agency whose aim is to provide for opinions on bioethical issues, published a specific investigation [5]. Later, two non-governmental agencies, the Hans Ruesch Foundation (HRF), and the Associazione Radicale Antispecista Parte in Causa (ARA), carried out further research [6,7].

In this paper, the authors critically reviewed data from the NBC, HRF and ARA reports and compared them with those emerging from their own investigation. The purpose of the analysis was to verify the legal compliance by academic faculties to inform students on their right to choose conscientious objection in situations where medical education involved the use of animals. In addition, the authors discuss the ethical implications that this sensitive issue enshrines.

## 2. Previous and New Investigations

### 2.1. The NBC Investigation

In 2009, the NBC published the report “Metodologie alternative, Comitati etici e obiezione di coscienza alla sperimentazione animale” (“Alternative Methods, Ethics Committees and Conscientious Objection to Animal Experimentation”) [5]. The report was based on replies to a questionnaire sent to 128 different scientific faculties at Italian universities and contained the following three questions:

Q1. Have your students been informed of their right to conscientious objection as stated in Article 3, Paragraph 5 of Law 413/93, which states: “All public and private establishments that legally carry out animal experimentation are obliged to inform all workers and students of their right to exercise conscientious objection with regards to animal experimentation. The establishments themselves are also obliged to set up a form for the declaration of conscientious objection to animal experimentation by the current Law”?
Q2. Have there been any cases of students making such a request?
Q3. Have you employed teaching methods that do not involve activities or interventions of animal experimentation to pass exams, as required by Article 4, Paragraph 3 of Law 413/93, which states: “University authorities shall make optional all laboratory activities where animal experimentation is foreseen. Within the start of the academic year that follows the coming into force of this Law, courses shall be offered that do not require activities related to animal experimentation as part of their final exam requirements. Universities’ student administration offices shall give maximum dissemination of students' right of conscientious objection to animal experimentation”?

As the NBC report stated, the duty to inform students of their right to animal CO has been partially ignored: 87 faculties out of 128 have informed their students, whereas 41 did not. Of the 41, 28 faculties justified the lack of dissemination of the law due to the absence of animal experimentation in their educational courses.

### 2.2. The Hans Ruesch Foundation (HRF) Investigation

After the NBC investigation, the HRF—an independent association that promotes the development of information on scientific activities involving animals—decided to promote and control the effective fulfillment of the faculties previously interviewed by the NBC.

First, the HRF asked the 41 faculties—that had previously declared to the NBC not to have informed their students—to comply with the information provisions set out by the Law 413/1993 via registered mail. Out of these 41 faculties, 14 responded that they subsequently would, whilst 27 faculties did not reply at all [6].

An additional audit of the faculties’ websites was carried out for all 87 “yes” responses to Q1 of the questionnaire sent out by the NBC. Furthermore, the HRF considered the presence of the text of Law 413/93 and the application form in the faculties’ website as a sign of compliance.

The results showed that only 10 of the cited 87 faculties published the text of Law 413/1993 on their websites.

Therefore, the HRF sent the 77 faculties that had not posted the information on their websites a formal request to comply with Law 413/93. Of these 77 faculties, 14 fulfilled the formal request and 62 did not. No further information about the remaining faculty is provided in the report.

The final results of the HRF report stated that only 41 out of 128 faculties were compliant (32%).

### 2.3. The “Nothing to Object?” ARA Campaign

The aim of the ARA, with its campaign titled “Nothing to object?” was to continue the HRF investigation [7]. Like the HRF, the ARA posited that the requirement for compliance with Law 413/1993 was the presence of information concerning the law and the declaration form on animal CO on the faculties’ websites.

Out of a sample size of 90 websites monitored, 81 did not provide any information. Therefore, the ARA sent these 81 faculties a formal request to comply with Law 413/1993. Of these 81 faculties, 39 subsequently complied with the regulations. In conclusion, out of the 90 faculties examined by the ARA, 48 (53%) were found to be compliant.

### 2.4. Our Own Investigation

In order to clarify the situation, we decided to carry out our own investigation into this matter. Along the lines of the HRF and ARA reports, we considered the presence of the text of Law 413/1993 and the declaration form on animal CO on the faculties’ websites as a criterion of “maximum dissemination”. With the expression “maximum dissemination”, we intended that complete information on animal CO would be clearly available and accessible, as well as easily consultable. Therefore, in 2016 we analyzed the websites of the 128 faculties previously examined by the NCB (through a questionnaire) and by HRF (through a check of the websites) using a double standardized methodology. First, accessibility to information was evaluated by consulting relevant sections, such as “Research”, “Courses”, “Services”, “Student Section”, and “Animal Legislation”. Next, expressions, such as “animal experimentation”, “conscientious objection”, “alternative methods” and “Law 413/1993” were used to fill in the search box of the websites. This consultation was not simple as the faculties were absorbed into research departments as a consequence of the reforms of the Italian university system governed by Law 240/2010, “Norme in materia di organizzazione delle università, di personale accademico e reclutamento, nonché delega al Governo per incentivare la qualità e l'efficienza del sistema universitario” (“rules on organization of Universities, academic staff and enrolment, as well as the government mandate to promote the quality and efficacy of the academic system”). For this reason, the majority of the faculties’ websites were replaced. In these cases, the department websites and “Course” sections were checked.

To sum up, 37 (28.9%) out of 128 faculties were found to be compliant. In 18 faculties (14.1%), the information was not sufficiently accessible to students, especially if they were not aware of its existence. More than half of the faculties (69) did not provide any information. In four cases, the websites were not functioning properly. The results of our investigation are summarized in Table 1.

## 3. Discussion

### 3.1. The NBC Investigation

The review of the NBC report highlighted an overlooked inaccuracy. In the report, only 10 faculties appeared to have responded positively to question Q2 “Have there been any cases of students making a request of conscientious objection?”; however, our analysis showed that Milano-Science and Lecce-Science had provided the answers “very rarely” and “rarely” rather than “no”, resulting in a total of 12 affirmative responses. Another problem derived from the NBC investigation regarded the use of the expression “animal experimentation”. As we have specified before, the NBC, according to Italian regulation, used this expression with a general meaning which included educational activities involving animals. This broader definition may have led some faculties to believe that Q1 referred solely to experimental practices involving animals, thus justifying the absence of information on their websites. The use of this generic and ambiguous term in the Italian system may have affected the awareness and response of those wishing to exercise their right to animal CO. Indeed, their choices may differ according to the different ways in which animals are used. It is worth noting that the first time the NBC questionnaire was submitted, teaching practices involving animals were allowed in universities. According to several universities and countries (including Germany, Czech Republic, Norway and Holland) [8], practices involving animals were banned by Law 26/2014 “in educational activities carried out in primary and secondary schools, as well as in university courses” (art. 5, point 2) [9]. The overall prohibition did not state which educational activities animal use was banned, rather, it included two exceptions: one for degree courses in veterinary medicine, and the other for postgraduate courses in medicine and in veterinary medicine.

### 3.2. The HRF Investigation

The results of the HRF investigation also revealed some inaccuracies. First, among the 62 faculties that had responded inappropriately “yes” to Q1 (as no information was present on their websites), two faculties (the Faculty of Pharmacy in Camerino and Faculty of Veterinary Science in Cagliari) were mistakenly counted as the Faculty of Pharmacy in Camerino had provided a response other than “yes” to Q1 and the Faculty of Veterinary Science in Cagliari was not included in the tables provided in the original HRF report. Furthermore, the wording “Pisa-Science” was probably used to indicate both the Faculty of Science in Pisa and the Faculty of Science of the “Scuola Normale Superiore” in Pisa. Finally, the HRF failed to provide any indication to responses to Q1 from three faculties: The Faculty of Medicine in Milan; the Faculty of Science in Varese; and the Faculty of Medicine in Catania.

### 3.3. Our Own Investigation and Overall Analysis

With regard to our investigation, a limiting factor was the criterion used to ensure maximum dissemination adopted by the HRF and ARA reports: the presence of the text of Law 413/1993 and the declaration form on animal CO on the faculties’ websites. The faculties’ websites could not be considered as the sole information source used by those faculties to inform students of their right to exercise animal CO. Indeed, faculties may use different delivery modes such as internal journals, information provided to students during enrolment, lectures, seminars, meetings and workshops. However, currently, faculty websites can be reasonably considered as the principal vehicle that university students consult. Students, in fact, regularly visit their faculty’s website to access information regarding their timetables, exam enrolments, teaching staff contact details, and other information necessary for their studies and their active participation in faculty life in general.

From a general comparative analysis of all the above cited reports, the results of the NBC, HRF and ARA show that in both 2009 and 2012, the level of compliance with Law 413/1993 remained at less than 50% of the faculties examined and non-compliance continued despite explicit requests. Our most recent report (2016) revealed serious inadequacies as information on Law 413/1993, where present, was often difficult to retrieve for those students not purposefully searching for it. Furthermore, information on animal CO was often positioned in sections that are not always visited by students. This situation of inadequacy may also be a consequence of the reforms introduced by Law 240/2010, which saw the migration of faculty websites to those of the new departments, resulting in a loss of information. In addition, Law 26/2014, which established a non-specific ban on animal experimentation for teaching purposes, may actually have contributed to reduce the awareness level of compliance with Law 413/1993. From this point of view, such an explanation provided by many faculties for their non-compliance with the duty of information would appear to justify the current situation. In addition, another reason for the continuing non-compliance by some faculties could derive from the misinterpretation of the poor wording of Law 413/1993.

### 3.4. Educational Animal Use and the Right to Animal Conscientious Objection

The authorized use of animals in educational activities brings up very controversial issues, especially considering it often involves the invasive use of animals. This is particularly questionable within the veterinary educational programs where in these cases, the ethical justification of the authorization is to promote medical knowledge useful to the betterment of all animals in society. However, this argument, which is grounded on the concept of the “right sacrifice of few for all”, raises several discussions. In general, the practical reason of using animals in education is to ensure that students are gain adequate skills that could not be achieved by alternative means, nevertheless, some studies also criticize this approach [10]. Furthermore, stressing the choice to invest in alternative methods constitutes a real path for the reduction of animal use. At an educational level, the reduction or the replacement of animal use can be carried out by using videos, computer simulations, inanimate models such as mechanical and plasticized specimens, and “ethically-sourced cadavers” from animals euthanized for medical reasons [11].

It is fundamental to remember that the right to animal CO should be granted to all students in the above-mentioned cases where animals are involved in teaching activities. Furthermore, it is worth noting that in addition to the cases where a ban is provided, students may be guided by teachers to use animals in the elaboration of their theses. As already clarified, an effective way to protect students’ right to opt for animal CO is to provide them with the specific information set out by the Italian legislation.

Finally, we believe that students have the right to choose animal CO regardless of whether they actually take part in activities involving animals. In fact, students may opt for animal CO simply to declare their individual ethical position regarding the possible use of animals in a particular setting (in this case, universities). Furthermore, as well as providing information regarding the possibility of exercising animal CO, universities could also inform students that, according to Law 14/2014, not all academic practices that involve animals and cause moral conflicts are subject to the right of conscientious objection. In particular, this right is not foreseen in animal slaughter methods and practices (not included in the animal experimentation practices set forth by Italian regulation) studied in veterinary science degrees [12]. Indeed, the role of a veterinary doctor is regarded as fundamental in guaranteeing food safety and animal welfare at the time of slaughter [13].

## 4. Conclusions

In spite of these limits, we believe that all investigations have made a valuable contribution to an issue that has been neglected for far too long. In fact, whilst opportunities to express conscientious objection to human issues such as voluntary termination of pregnancy and to medically-assisted procreation have benefited from extensive information campaigns, the same cannot be said of animal CO.

Such scarce attention reveals a lack of sensibility to animal ethics issues that contrasts with the principles of animal ethics in the so-called 3Rs (Reduction, Refinement, Replacement), which underpin European law dedicated to protecting animals used in scientific activities [14,15]. Respect of these principles plays an important role in raising personal awareness amongst students on ethical issues [11,12,13,14,15,16,17]. As seen in the literature [18], students who are encouraged to contemplate ethical issues can actively contribute to the development of rules that respect different ethical points of view.

Appropriate strategies should be developed so that the issue of animal ethics and the right to exercise animal CO receives adequate attention.

Careful monitoring of compliance by faculties regarding their duty of information could be carried out by bioethical committees such as the cited NBC; or by animal welfare bodies established by Law 26/2014 under Directive 2010/1963/EU with the specific purpose to safeguard animal welfare, as well as by the Italian Ministry of Education, University and Research. Additionally, the Ministry should officially reprimand non-compliant faculties that do not uphold their obligations of information and reward compliant faculties with special funds. These benefits could be released with the purpose to balance any additional costs that universities would incur in order to ensure adequate alternative scientific activities and programs.

With regard to the scientific literature, we hope for greater attention on animal issues and the development of further investigations such as a direct survey on students’ knowledge in this field to better understand the reasons for Italian non-compliance with national law provisions.

## Figures and Tables

**Table 1:** Results of our investigation about the information on the Law 413/1993 on Faculties’ websites.

Law Fulfillment	Information on Law	No. of Websites	%
Adequate	Easy *website consultation* ^1^ and effective *search box* ^2^	32	29%
	Easy *website consultation* only	5	
Inadequate	Poor *website consultation* and effective *search box*	3	14%
	Poor *website consultation* only	4	
	Effective *search box* only	11	
None	Absence of any information	69	54%
Not evaluable	Run time error of the website	4	3%
		128	100%

^1^ Accessibility to the information by consulting the website; ^2^ Accessibility to the information by using the search box.
